# Is there a relationship between pain intensity and postural sway in patients with non-specific low back pain?

**DOI:** 10.1186/1471-2474-12-162

**Published:** 2011-07-15

**Authors:** Alexander Ruhe, René Fejer, Bruce Walker

**Affiliations:** 1 Murdoch University, Praxis fuer Chiropraktik Wolfsburg, Wolfsburg, Germany; 2 Chiropractor, Research Department, Spine Centre of Southern Denmark, Hospital Lillebaelt and University of Southern Denmark, Middelfart, Denmark; 3 School of Chiropractic and Sports Science, Murdoch University, Murdoch, Western Australia, Australia

## Abstract

**Background:**

Increased center of pressure excursions are well documented in patients suffering from non-specific low back pain, whereby the altered postural sway includes both higher mean sway velocities and larger sway area. No investigation has been conducted to evaluate a relationship between pain intensity and postural sway in adults (aged 50 or less) with non-specific low back pain.

**Methods:**

Seventy-seven patients with non-specific low back pain and a matching number of healthy controls were enrolled. Center of pressure parameters were measured by three static bipedal standing tasks of 90 sec duration with eyes closed in narrow stance on a firm surface. The perceived pain intensity was assessed by a numeric rating scale (NRS-11), an equal number of patients (n = 11) was enrolled per pain score.

**Results:**

Generally, our results confirmed increased postural instability in pain sufferers compared to healthy controls. In addition, regression analysis revealed a significant and linear increase in postural sway with higher pain ratings for all included COP parameters. Statistically significant changes in mean sway velocity in antero-posterior and medio-lateral direction and sway area were reached with an incremental change in NRS scores of two to three points.

**Conclusions:**

COP mean velocity and sway area are closely related to self-reported pain scores. This relationship may be of clinical use as an objective monitoring tool for patients under treatment or rehabilitation.

## Background

Increased postural sway is well documented in patients suffering from non-specific low back pain (NSLBP) [1] and a variety of theories exist regarding the effect of NSLBP on body sway. Postural control mechanisms are believed to be affected by damage to sensory tissues in the lumbar spine and trunk [2]. This deterioration of proprioceptive information reduces the accuracy of the sensory integration processes resulting in an imprecise estimation of the center of mass position [3], thereby inhibiting compensatory center of pressure (COP) shifts.

Acute "pain interference" [4] has also been proposed as a possible cause with discharge from high-threshold nociceptive afferents in the low back interfering with spinal motor-pathways [5] and the motor cortex [6]. In addition, pain may cause an increased pre-synaptic inhibition of muscle afferents [7] and affect the central modulation of proprioceptive spindles of muscles [8], thereby causing prolonged latencies by a decrease in muscle spindle feedback.

As outlined in our systematic literature review [1], several factors such as age [9-11], gender, weight [12], and height [13] have been shown to exhibit a significant effect on postural sway. The aim of this study is to investigate whether COP excursions are also affected by pain severity and pain duration and if so, to further describe this relationship. This relationship is worthy of investigation as it may show clinical significance for the application of COP measures.

To our knowledge, this is the first study to investigate this clinical question with a best practice experimental setup and also the first to comprehensively assess the relationship between pain and COP excursions over a wide spectrum of pain scores when compared to healthy controls.

## Methods

### Subjects

We aimed at enrolling around 80 participants for both symptomatic and control group. Previous sample size calculations for a group of controls and symptomatic patients with an NRS-11 score of 5.0 ± 2.1 using an Altman Nomogram [14] suggested recruitment of around 50 symptomatic and healthy participants each. We decided to exceed this number in order to compensate for potential dropouts.

All new patients entering a private chiropractic clinic in Wolfsburg, Germany were asked on the phone whether they would take part in this study. The healthy controls were friends and partners of already enrolled participants and were initially approached by them regarding the possibility of participation. If they displayed interest they were asked to contact the clinic for further details. After verbal and written information had been given, the subjects consented to participate in this study, which was approved by the Murdoch University Human Research Ethics Committee (Approval 2010/173).

The cut-off age for both controls and symptomatic individuals was 50 years, as after that age related impairments to postural stability could not be excluded [9-11].

Inclusion criteria for the symptomatic participants were NSLBP of any duration and the presence of pain ≥ 2 on the NRS-11 scale on the day of the postural sway recordings. Participants were excluded if the pain went below the gluteal fold, there were positive nerve root findings, serious spinal deformities, any condition that might affect balance (e.g. whiplash associated disorder or vestibular pathologies) or previous significant injuries such as traumatic damage to the spine or spinal surgery. No pain medication was allowed within 24 hours prior to the recordings. Participants were also excluded if they were unable to perform the postural sway recording either due to pain or other reasons. We aimed at enrolling around 10 patients for the 9 pain intensity groups (NRS 2-10).

For the purpose of this study, healthy was defined as the absence of any self-reported neurological or musculoskeletal impairments, pain or disability for a minimum of 6 months prior to the time of evaluation. Specifically, individuals with a history of low back pain within 6 months or previous injury to the neck or lower extremities, any known balance problems or the usage of medication associated with pain suppression or altered sensory perception were excluded. The physical examination of the control group must also have ruled out any back or extremity complaints or significant biomechanical impairments that might influence the measurements.

### Procedures

Prior to the COP measurements, a physical examination was conducted on all participants by two experienced and trained chiropractors (TB and AS) who were otherwise not involved in the study. This procedure aimed to assess whether the volunteers met the criteria for their respective group and met the physical demands of the study. The NSLBP participants were further asked to describe their pain intensity at the time of recording by means of an NRS-11, a rating scale ranging from 0 (no pain) to 10 (worst possible pain) [15].

The experimental setup was based on an earlier literature review where a best practice setup for obtaining reliable COP data was published [16]. Accordingly, trials were conducted with eyes closed as the data obtained shows higher reliability than with eyes open. We further considered that the loss of visual input would prove an additional challenge to the balance system. In this way deficits in proprioception may be more easily detected and the discriminative value of the measurement between healthy controls and symptomatic NSLBP participants enhanced.

The system used for this study was a Metitur Good Balance GB300^® ^CE (Metitur Oy, Finland). Signals were sampled at 100 Hz, amplified and converted from analogue to digital. High frequency noise was reduced by a low-pass filter with a cut-off frequency of 10 Hz.

Mean velocity (mVel) was chosen as the main COP parameter as this has consistently shown to be both reliable [16] and discriminative for NSLBP [1]. It is described by taking the total distance of the COP path travelled in the respective direction and dividing it by the sampling duration. In addition, the 90% circle diameter was included to offer a broader spectrum of analysis. This parameter refers to the diameter of a circle containing 90% of the COP path travelled over a given time.

The participants were asked to remove their shoes and stand upright on the forceplate with their eyes closed, the head erect and their arms hanging loosely by their sides. The foot position was narrow stance with toes and heels touching. For the duration of the recording, the participants were further instructed to "stand as still as possible" [17].

Three successive trials of 90 seconds duration each were conducted with a preceding 5 sec adaption period that was not recorded. Rest periods of 60 sec were provided between each trial during which the participants were allowed to sit down while maintaining their original foot position on the forceplate. All participants were asked afterwards whether pain or discomfort may have influenced their balance performance.

All tests were conducted in a quiet room with normal temperature. The forceplate was calibrated prior to the recordings and further underwent an automatic calibration check before each trial.

### Data analysis

#### Age effects

To test if postural sway is influenced by age [9-11], the healthy participants were subdivided into two age ranges (20-35 and 36-50 years) and subsequently compared to see if they statistically differ from each other. If, however, our study showed no significant differences, the age groups were to be combined for further analysis to reduce the risk of type-II error.

#### Reliability

To test the reliability of the COP measures for this experimental setup for both controls and pain sufferers, the two-way random-effect intra-class correlation coefficient (ICC2,k) as described by Shrout and Fleiss [18] was computed using absolute agreement. For the purpose of this study it was interpreted using the following criteria: 0.0-0.39 poor, 0.40-0.59 fair, 0.60-0.74 good and 0.75-1.00 excellent [19]. In addition, the 95% confidence intervals (CI) and the standard error of measurement (SEM) [20] were calculated.

#### Relationship between pain intensity and postural sway

We also tested the assumptions of homogeneity of variance (Levene statistic) and normality, where Shapiro-Wilk test was conducted for all independent variables and the dependent variables separately per pain group. The COP data was further analyzed using the Games-Howell test. Means, standard deviations (SDs) and 95% confidence intervals (CIs) were calculated for all dependent variables.

Stepwise univariate regression analysis was conducted to assess for the possible effect of each of the following variables: age, gender, weight, height, pain intensity and previous pain duration on COP mVel and 90% circle diameter. This was followed by a multivariate regression analysis where independent variables that showed a significant effect during univariate analysis were included.

To investigate the appropriate form of regression analysis, the SPSS Curve Estimation function was applied to scatter plots for variables stated above (independent variables) and the COP parameters (dependent variables). In addition, collinearity diagnostics were applied. The level of statistical significance was set at *p *≤ 0.05.

All data were exported to PASW^® ^Statistics 18 (SPSS Inc, 2009) for statistical analysis.

## Results

### Subjects

Eighty-two individuals suffering from NSLBP initially volunteered to participate in this study. We did not reach our target number of at least 10 NSLBP participants for NRS scores 9 (n = 2) and 10 (n = 0) and therefore only included NRS scores 2-8 with 11 NSLBP participants each. Five symptomatic participants were excluded as they exhibited severe pain (n = 4) or an antalgic posture (n = 1) when standing and were unable to complete the tests. This left a total of 77 NSLBP sufferers (37 females, 45%) to which a matching number of healthy controls were enrolled. All participants were able to complete the trials without difficulty and did not report increased pain or discomfort during the COP recordings. The characteristics of the participants are shown in Table 1.

**Table 1:** Demographic and functional characteristics

	NSLBP Age 20-35 (n = 32)	Healthy controls Age 20-35 (n = 36)	Statistical difference p-value	NSLBP Age 36-50 (n = 45)	Healthy controls Age 36-50 (n = 41)	Statistical Difference p-value
Age (years)	28.9 ± 4.7	29.8 ± 4.4	0.89	44.1 ± 4.3	43.5 ± 5.5	0.67
Height (cm)	178.0 ± 6.6	177.2 ± 7.4	0.36	179.2 ± 7.6	176.9 ± 6.9	0.37
Weight (kg)	77.6 ± 9.5	77.3 ± 11.7	0.47	80.8 ± 12.8	76.9 ± 8.8	0.71
BMI	24.3 ± 2.7	24.9 ± 3.9	0.60	25.1 ± 2.9	24.5 ± 1.9	0.11
NRS-11 (0-10)	4.9 ± 1.9	N/A	N/A	5.1 ± 2.1	N/A	N/A
Previous pain duration (weeks)	19.9 ± 33.6	N/A	N/A	18.7 ± 30.5	N/A	N/A

Values are mean ± SD

### Age groups

Both age groups had a similar number of healthy participants with n = 36 for 18-35 yrs and n = 41 for 36-50 yrs. As there was no statistically significant difference in COP measures between the two groups (Table 2), the data were combined and analyzed for the control group as a whole.

**Table 2:** Comparison of COP data between the age groups

COP parameter	Healthy controls 20-35 yrs (n = 36)	Healthy controls 36-50 yrs (n = 41)	Statistical difference p-value
mVel ML (mm/s)	11.8 ± 2.5	12.0 ± 2.7	0.28
mVel AP (mm/s)	9.1 ± 2.7	9.5 ± 2.1	0.27
90% circle diameter (mm)	11.6 ± 2.8	12.0 ± 2.4	0.21

Values are mean ± SD

AP: antero-posterior, mVel: mean velocity, ML: medio-lateral

### Reliability

With three recordings being averaged from the both healthy controls and symptomatic participants, the included COP parameters reached good reliability throughout (Table 3).

**Table 3:** Reliability of COP measures

COP parameter	NSLBP (n = 77)			Healthy controls (n = 77)		
	
	ICC2,k	95%CI	SEM	ICC2,k	95%CI	SEM
mVel ML	0.85	0.79-0.99	0.96	0.89	0.73-0.97	0.89
mVel AP	0.83	0.76-0.88	0.86	0.85	0.63-0.96	0.96
90% circle diameter	0.71	0.61-0.79	1.29	0.69	0.57-0.77	1.44

AP: antero-posterior, mVel: mean velocity, ML: medio-lateral

### Relationship between pain intensity and postural sway

As a general trend, a steady linear increase in mVel AP/ML and 95% circle diameter direction can be observed. Levene's Tests showed no homogeneity of variance (*p *≤ 0.018) while Shapiro-Wilk test indicated a normal distribution of the independent and dependent variables (p ≥ 0.11).

**Figure 1:**
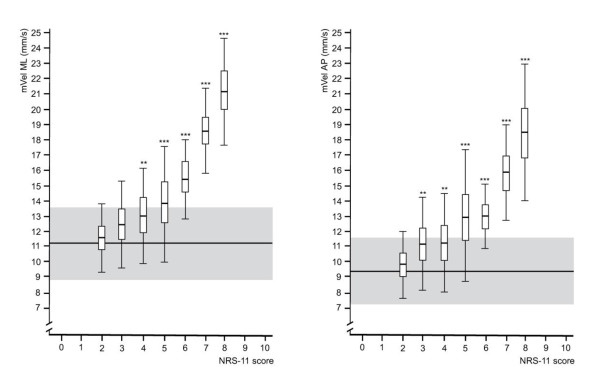
**Relationship between pain intensity and mean sway velocity in AP and ML**. The horizontal line and the grey area indicate the mean score of healthy controls and the standard deviations respectively. The vertical lines indicate standard deviations; the boxes show mean and 95% CIs respectively. Levels of significance compared to controls: * *p *≤ 0.05, ** *p *≤ 0.01, *** *p *≤ 0.001.

Compared to healthy controls, a significant difference (*p *≤ 0.01) in mVel was present in NSLBP participants beginning at an NRS score of 3 in ML direction. In AP direction, statistical significance (*p *≤ 0.05) was also reached at a pain intensity of 3 with an increase in significance from 5 to 8 (*p *≤ 0.001) (Figure 1).

**Figure 2:**
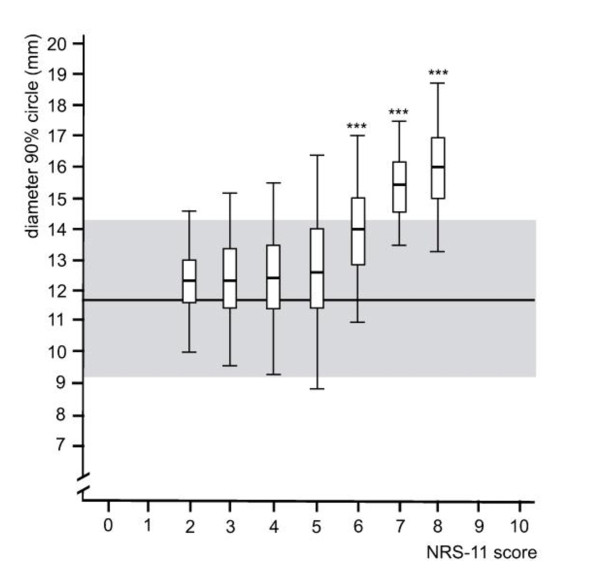
**Relationship between pain intensity and 90% circle diameter**. The horizontal line and the grey area indicate the mean score of healthy controls and the standard deviations respectively. The vertical lines indicate standard deviations; the boxes show mean and 95% CIs respectively. Levels of significance compared to controls: * *p *≤ 0.05, ** *p *≤ 0.01, *** *p *≤ 0.001.

Compared to healthy controls, a significant difference in 90% circle diameter was only present at NRS scores of 6, 7 and 8 (*p *≤ 0.001) (Figure 2).

The differences in postural sway between pain scores as assessed by Games-Howell are presented in Tables 4 and 5. With regards to mVel differences between the individual pain scores, significance was reached at lower NRS scores in ML compared to AP direction (Table 4).

**Table 4:** Sway differences between NSLBP participants and pain free controls using NRS-11 scores for mVel AP and ML

NRS-11 Score												
8	***	***	***	***	***	***	***	***	***	***	**	*
7	***	***	***	***	***	***	***	**	***	***		
6	***	***	***	*	***	*	*	n.s.				
5	**	***	n.s.	n.s.	n.s.	n.s.						
4	*	**	n.s.	n.s.								
3	n.s.	n.s.										
2												
	
	ML	AP	ML	AP	ML	AP	ML	AP	ML	AP	ML	AP
	**2**		**3**		**4**		**5**		**6**		**7**	
	**NRS-11score**											

n.s.: not significant (p > 0.05)

Levels of significance: * p ≤ 0.05, ** p ≤ 0.01, *** p ≤ 0.001

**Table 5:** Sway differences between NSLBP participants and pain free controls using NRS-11 scores for 90% circle diameter

8	***	***	***	**	*	n.s.
7	***	***	***	**	*	
6	*	n.s.	*	n.s.		
5	n.s.	n.s.	n.s.			
4	n.s.	n.s.				
3	n.s.					
2						
**NRS-11** **Score**	**2**	**3**	**4**	**5**	**6**	**7**

n.s.: not significant (*p *> 0.05)

Levels of significance: * *p *≤ 0.05, ** *p *≤ 0.01, ***p ≤ 0.001

Finally, the relative differences between pain scores for the parameter 90% circle diameter are demonstrated in Table 5. The same trend as seen with mean sway velocity can be observed. However, at pain intensities 2 and 3, significant differences between pain scores are present at larger intervals (3 NRS scores compared to 1-2 at mVel ML/AP).

### Regression analysis

The SPSS Curve Estimation function showed that a linear relationship was the most suitable line of fit (p ≤ 0.001). Hence, linear regression was used for further analyses of the data. No co-linearity between the variables was determined.

The univariate regression analysis included the variables gender, age, weight, height, previous pain duration and pain intensity. With the exception of previous pain duration, all other independent variables exhibited a significant effect on mVel AP/ML and 90% circle diameter and were consequently included in the multivariate analysis. This further analysis showed that only pain intensity exhibited a significant effect on the selected COP parameters.

For mean velocity and pain intensity, the regression analysis was a reasonably good fit, describing 53.0% of the variance in mVel ML and 40.0% in mVel AP (R^2^adj = 51.0% and R^2^adj = 38.4% respectively), the overall relationship was highly significant in both ML and AP direction (F = 40.8, *p *< 0.001 and F = 24.9, *p *< 0.001 respectively). Mean sway velocity increased by 1.53 mm/s for every extra pain level in ML, and by 1.27 mm/s for every extra pain level in AP direction.

The regression analysis for the parameter 90% circle diameter and pain intensity was a poor fit, describing just 18.7% of the variance in circle diameter (R^2^adj = 16.5%). The overall relationship, however, was highly significant (F = 8.6, *p *< 0.001). The 90% circle diameter of the COP excursion increased by 0.6 mm for every extra pain level.

## Discussion

We were unable to enroll a sufficient number of NSLBP participants for all pain intensity groups to allow analysis of all 10 NRS scores. This may be explained by the fact that patients with NRS scores of 9 and higher are not commonly encountered in a chiropractic practice as the potential severity of the condition warrants medical attention instead.

We were able to demonstrate a linear relationship between pain intensity and postural sway velocities in both AP and ML direction as well as for the parameter 90% circle diameter. This is in agreement with a general observation by Lihavainen et al. [21] who conducted a similar study in a geriatric population. They did not, however, investigate postural sway related to the individual pain scores but reached their conclusions based on a subdivision into mild or moderate/severe pain only.

Even though an increased sway velocity started at a lower pain score in the AP direction, the overall difference compared to healthy controls was similar to that in the ML direction. On the other hand, the ML sway velocity increased at a faster rate. In addition, this study confirms the altered postural sway characteristics previously reported in a systematic review of NSLBP sufferers [1]. The review noted higher COP mVel values (particularly the AP direction) and a larger sway area compared to healthy controls was described.

The non-overlapping 95% CIs associated with NRS scores at higher pain intensities, particularly with mVel AP/ML, are surprising and may be attributable to our standardized experimental setup and selection of participants. Such a clear subdivision appears unlikely at first sight due to the inherently varying pain perception between individuals.

As the 90% circle diameter is exclusively used with the Metitur system, it is not possible to put the respective results into context. However, it corresponds to the various parameters applied in the literature to describe COP sway area and may therefore offer at least limited comparability.

Our data, however, does not allow for an explanation of the underlying mechanism of the observed pain associated alterations in COP sway velocity. However, as previous pain duration did not exhibit a significant effect on postural sway as pain intensity has, this may suggest that pain interference [4] may be the determining factor. Neuro-physiological changes, on the other hand, are rather dependent on pain duration and therefore a significant time effect would have been expected. Future studies assessing postural sway before and after acute pain stimulation or using analgesics in chronic and acute NSLBP participants may add valuable information in this respect.

Furthermore, as no other studies have looked into the relationship between a broader range of pain intensities and COP measures it is not possible to compare our results.

At lower and medium pain intensities there was no apparent change in the COP parameters. This may be due to participants finding it difficult to decide on their "true" score, NRS-5 for example shows the widest standard deviations across all parameters. This may therefore explain why no statistically significant differences were observed between lower pain scores (NRS 2-4) for most parameters and may account at least partially for the variability in the associated COP measurements. However, as the confidence intervals across all pain scores remain fairly consistent, the variability of the postural sway measurements most likely reflects individual variations within the COP excursions. The results also suggest that the neurological alteration previously described [4-8] may only have an impact on COP measures at medium to high intensities (i.e. NRS ≥ 5).

In contrast to other studies [9-13], we could not demonstrate any significant effect of age, height, weight or gender on COP excursions in the patient group. This may be attributed to the demographics and physical characteristics of the participants as well as our COP measurement protocol. Our results were derived using a protocol based on best evidence [16], nevertheless future studies are needed to confirm these findings using the same protocol.

### Clinical considerations

The COP measurement protocol used in this study may in future be suitable as an objective outcome measure for clinical monitoring purposes. However, the results are unidirectional in that increasing pain was associated with increasing postural sway. We have not established that decreasing pain leads to a decreasing postural sway.

Secondly, given the linear relationship between pain intensity and, for example, mVel, a clinically significant decrease of two points on a pain NRS [20] is equivalent to a reduction in mean sway velocity of 3.6 mm/s in ML and of 3.0 mm/s in AP direction. These changes lie between 1 and 2 standard deviations from the mean. It remains to be seen if such a reduction is also clinically significant.

In addition, this study indicates that any future sample size calculations for COP measurements involving pain sufferers should be considered in the light of the respective perceived intensity. Depending on the research purpose, the inclusion criteria may focus on those with NRS-scores of 5 or higher to reach significance compared to controls more readily.

The results may also cast a new light on the interpretation of studies that reported no significant differences in postural sway between symptomatic individuals and healthy controls. In those instances (e.g. Brumange et al. [22] and Mok et al. [23]), these observations may be attributable to the low perceived pain intensities of the NSLBP participants enrolled.

There is evidence that higher COP sway is associated with a higher risk of falling in the elderly [24] and sustaining injuries as a consequence, although this is subject to debate [25,26]. Our results did not include geriatric participants and therefore cannot be generalized to that population, however our data may nevertheless underline the importance of suitable pain control in elderly pain sufferers to avoid falls.

In addition, as pain interference appears a likely underlying mechanism, the focus of a rehabilitative approach in pain sufferers with increased COP excursions should be on pain reduction rather than proprioceptive training.

Future studies may also show a role for COP measurements as part of a suite of other procedures to identify malingerers. Even if the individual is aware that pain is associated with greater COP excursions, a study with pseudo-malingerers showed that imitating pain related sway pattern is difficult at best and the average results for sway velocity and sway area greatly exceeded those expected from a real pain sufferer [27].

### Strengths and Limitations

The major strength of this study is in its best practice experimental setup which ensured reliable data collection. Our inclusion and exclusion criteria further prohibited our overall results from being affected by demographic or anthropometric factors.

In this cross-sectional study the subjective nature of pain perception and therefore pain rating may have influenced the results. In addition, pain perception between younger and older NSLBP participants varies and a decrease in pain perception in geriatric individuals has been described [28]. Although this does not affect our sample groups with a cut-off age of 50 yrs, it nevertheless prohibits our results to be generalized to elderly patients.

While significant differences in postural sway compared to healthy controls could be demonstrated in our patient population, the overall number of participants per NRS score was still comparably small. Our results are therefore prone to be affected by extreme COP measures. Other sample groups with identical NRS scores may therefore show varying results. However, we expect the linear trend to be preserved. Similar studies with an identical experimental setup and larger sample sizes should be conducted to confirm our results.

## Conclusions

Despite the subjective nature of pain perception and the unclear causative factors, the results of this study show that in adults (18 and 50 years) with NSLBP, increasing COP sway velocity increases linearly with increasing perceived pain intensity greater than 4 on an NRS scale. This trend, while less obvious, is also apparent for the parameter 90% circle diameter.

## Competing interests

The authors declare that they have no competing interests.

## Authors' contributions

AR carried out the COP measurements, conducted the statistical analysis and drafted the manuscript. RF and BW participated in the study design and assisted in drafting the manuscript. All authors read and approved the final manuscript.

## Pre-publication history

The pre-publication history for this paper can be accessed here:

http://www.biomedcentral.com/1471-2474/12/162/prepub
